# Stimulating collaboration between human and veterinary health care professionals

**DOI:** 10.1186/s12917-017-1072-x

**Published:** 2017-06-13

**Authors:** Björn G.M. Eussen, Jaap Schaveling, Maria J. Dragt, Robert Jan Blomme

**Affiliations:** Center for Leadership and Management Development, Nyenrode Business University, P.O. Box 130, 3620 AC Breukelen, Netherlands

**Keywords:** One health, Social dilemma, Collaboration, Veterinarians, Physicians, Knowledge sharing, Common Ingroup Identity Model

## Abstract

**Background:**

Despite the need to control outbreaks of (emerging) zoonotic diseases and the need for added value in comparative/translational medicine, jointly addressed in the One Health approach [One health Initiative (n.d.a). *About the One Health Initiative.* http://www.onehealthinitiative.com/about.php. Accessed 13 September 2016], collaboration between human and veterinary health care professionals is limited. This study focuses on the social dilemma experienced by health care professionals and ways in which an interdisciplinary approach could be developed.

**Results:**

Based on Gaertner and Dovidio’s Common Ingroup Identity Model, a number of questionnaires were designed and tested; with PROGRESS, the relation between collaboration and common goal was assessed, mediated by decategorization, recategorization, mutual differentiation and knowledge sharing. This study confirms the Common Ingroup Identity Model stating that common goals stimulate collaboration. Decategorization and mutual differentiation proved to be significant in this relationship; recategorization and knowledge sharing mediate this relation.

**Conclusions:**

It can be concluded that the Common Ingroup Identity Model theory helps us to understand how health care professionals perceive the One Health initiative and how they can intervene in this process. In the One Health approach, professional associations could adopt a facilitating role.

## Background

To control outbreaks of (emerging) diseases at an early stage, effective collaboration between human and veterinary healthcare professionals is essential [1]. However, to date, collaboration has taken place only on a very limited scale [1–6]. For this reason, One Health, an interdisciplinary approach addressing the connections between health care for humans, animals and the environment and focused on the elements biomedical research, enhanced public health efficacy, an expanded scientific knowledge base and improved medical educational and clinical care in which human and veterinary healthcare and other stakeholders work together [7], is placed high on the agendas of organizations such as the WHO, the European Commission’s Directorate-General for Health and Consumers, USA Centers for Disease Control and Prevention [CDCP] and Worldbank [1, 8–10].

Because more than three-quarters of all the infections seen in humans originate in animals [1], there is great and widespread interest in the effective collaboration between human and veterinary healthcare professionals as a means to halt outbreaks of infections at an early stage. The outbreak of Severe Acute Respiratory Syndrome (SARS), for instance, which was not even known to be an animal-transmitted infection when it first occurred, is estimated to have caused a loss of as much as US$40 billion in terms of Gross Domestic Product (GDP) worldwide [11]. Other examples include the recent outbreaks of avian influenza and Q fever in the Netherlands, which have shown that serious outbreaks can have major consequences for human and animal health [12]. Areas where close cooperation would be fruitful because they share the same goals include combatting and controlling zoonoses and emerging zoonoses; here, the interdependence of the two fields requires an integrated approach. In efforts to reach mutual goals, an interdisciplinary exchange of knowledge between the two professional groups can lead to a sharing of domain-specific expertise and experience, thus enabling experts to identify possible zoonotic infections and to fight them with the help of appropriate control measures at earlier stages [2]. Another advantage of collaboration between these two groups of healthcare professionals, besides the more adequate fight against infectious diseases, is the exchange of knowledge on the treatment of diseases [2] leading to major healthcare cost savings [6, 11] and to new scientific insights [7]. In order to better understand the mental motivation of health care professionals with respect to the One Health approach, this study used psychosocial concepts such as social dilemma, (group) identification and category thinking.

### Collaboration as a social dilemma

Collaboration can be defined as “any action which is intended to benefit others, regardless of whether the actor also benefits in the process” [13]. However, collaboration can also fail to take place: in human and veterinary healthcare, cooperation between professionals can fail to materialize because there is a social dilemma, for instance. To illustrate, there are situations in which a non-cooperative course of action is (at times) tempting for each individual in that it yields superior (often short-term) outcomes for the individual himself or herself [13]. For example, it is commonly accepted that professionals have a constant and permanent responsibility for their patients, who expect care and action in the short term. A healthcare professional may therefore view collaboration, which may (at times) exceed or influence his own day-to-day operations, as not sufficiently important, at least for the short term. But if everyone pursues this non-cooperative course of action, then everyone will ultimately be worse off (often in the longer term) than would have been the case if everyone had collaborated.

Collaboration between human and veterinary healthcare professionals can be characterized as a social dilemma. It is often thought that a lack of time is the main reason why healthcare professionals feel they have so little psychological room for greater collaboration. Fleuren, Wieferink and Paulussen [14], however, show that healthcare professionals actually have other reasons than time; what lies at the heart of their limited cooperation is a lack of clarity as to why collaboration should be organized in the first place. The individuals concerned do not always have a sufficiently well-defined idea of the benefits offered by increased contact and collaboration, even though collaboration is highly desirable both for the sector itself and for society as a whole [2]. In this regard and in any event, the (long-term) awareness of sharing a common goal is of crucial importance.

Collaboration is stimulated if the (perceived) benefits outweigh the individual arguments or circumstances of the healthcare professionals concerned. This type of calculation, or dilemma, can lead to cooperation ‘for the public good’. Considering its objectives, One Health can be seen as a public good dilemma, because “public good dilemmas require individuals to make an active contribution to establish or maintain a collective good, such as building a local bridge or joining a social movement” [13]. However, in terms of making room for the bigger collective goal alongside their responsibilities related to the day-to-day care of their own patients, human and veterinary healthcare professionals often see insufficient added value [14], even though a greater awareness of the added value associated with collaboration would ultimately result in improved care [5, 15, 16].

Greater awareness of the added value brought about by collaboration on the part of healthcare professionals is consistent with the social desire for more intensive collaboration between human and veterinary healthcare [1, 8–10, 12]. These two groups of healthcare professionals speak the same ‘language’ and should therefore be able to understand each other well [17]. Despite the fact that, broadly speaking, both groups followed similar training programmes [18, 19] and perform similar clinical procedures, there is hardly any exchange of knowledge and experience [5]. In addition, a clear focus on common goals is lacking, and collaboration between human and veterinary healthcare professionals remains limited at present [5, 6, 20]. So far, collaboration has only taken place in a limited number of research areas and only occasionally during outbreaks of emerging diseases [1, 4]. Closer examinations have revealed that the limited scale of collaboration is due, among other things, to mutual prejudices [2] and psychological barriers between the parties concerned [5]. Such mutual judgements and prejudices may disrupt the development of collaboration, but research into these phenomena has so far remained very limited. The present study is aimed to help fill that gap.

### Common ingroup identity theory

In order to gain a better understanding of what stimulates cooperation among healthcare professionals, we used the Common Ingroup Identity Model developed by Gaertner and Dovidio [17]. This theory provides insight into the relationship between individual perceptions and behaviours towards groups and lists possible causes which may influence these. According to the Common Ingroup Identity Model, sharing a common goal affects the degree of collaboration, but this relationship is also influenced by the perception of this degree and the means of categorization [17, 21]. The Common Ingroup Identity Model [17] focuses on the individual’s perception with regard to the group. In essence, the model states that members who see themselves as belonging to a larger, common whole consciously classify themselves within that larger whole, as a result of which prejudices between groups or communities decrease.

According to Gaertner and Dovidio, collaboration is influenced by characteristics (in this study qualified as the common goal) which play a role in the individual perception of the situation: does the individual perceive the existence of a single overarching group, several groups or subgroups, or no group at all? If individuals feel that they belong to a group, in this case ‘healthcare’, this will lead to more positive thoughts, feelings and behaviours among the individuals in the groups concerned [17]. As explained above, when group members see themselves as part of a larger whole and when they classify themselves within this structure, prejudice between groups or communities is reduced. According to Kramer and Brewer [22], people within a group or an overarching whole are prepared to share communal resources and other supplies, but they will develop resistance if these have to be shared with others *outside* the group. Still, a change occurs if these outsiders can be placed within a perceived larger whole. The realization that there is in fact a larger whole means that the positive thoughts, feelings and behaviours (such as the sharing of resources and information) which would normally be reserved for the individual’s own familiar group are extended to members of other communities who also belong to the larger overarching whole. Common endeavour and classifying thus go hand in hand, leading to feelings of ‘us’ rather than feelings of ‘us versus them’. In other words, it depends on how an individual sees his group or subgroup within a larger whole [17].

Using the Common Ingroup Identity Model, we quantitatively assessed healthcare group interrelationships in order to gain insight into the contributions towards cooperation that can be made by means of a common goal for healthcare professionals formulated via their perception of group formation. In the past, the Common Ingroup Identity Model was researched primarily in experimental studies, with a main focus on perceived group formation [23]. A limitation of the model is that the duration of the effect of an intervention is unclear, as is the reduction of bias [24]. The current study will not only indicate whether the Common Ingroup Identity Model is useful for the respective groups of healthcare professionals, but it will also quantitatively assess the relationships between the common goal and collaboration in combination with associated mediating factors. In this way, the study will contribute to further theoretical development in terms of validation as well as to the quantitative usefulness of the Common Ingroup Identity Model. It will also examine whether the exchange of knowledge is an additional trigger for collaboration once healthcare professionals have become aware of the common goal.

### Collaboration and common goal

In the social dilemma referred to earlier, where there is insufficient awareness of the possible advantages of collaboration, and in this case human and veterinary collaboration, crucial factors include the reasons why healthcare professionals place themselves in a particular category and identify with their ‘own’ professional group [2, 3, 5]. It is ‘natural’ for people to engage in social categorization: the brain is hard-wired to think in terms of categories, and categories form the basis for standard judgements and prejudices [25]. The advantages of social categorization are that individuals know where they stand and what is expected of them, and that a group or community contributes to a feeling of (social) well-being [17]. The same is true of human and veterinary healthcare professionals, although a certain distance is maintained between them [2, 5]. After all, what is qualified by the terms ‘human’ and ‘animal’ is placed in different categories. This is illustrated by the dichotomy between the two professional groups, established on the basis of typical activities, with ‘human patient’ being contrasted with ‘animal patient’, and hence on the basis of category-based thinking [2]. Still, mutual contact alone does not lead to productivity or better joint results; for good results, interdependence is necessary [26, 27]. If individuals perceive a common goal, in this case ‘improving care through One Health’, then according to the Common Ingroup Identity Theory this can be expected to lead to more positive thoughts, feelings and behaviours between individuals in the groups concerned [17], because there will then be more perceived interdependence between the two groups. According to the Common Ingroup Identity Theory, the awareness of a larger whole or a common ingroup, in this case a joint responsibility for improving care, will lead to the extension of positive thoughts, feelings and behaviours (such as the sharing of resources and information) that were traditionally reserved for the individual’s own familiar group to members of other communities who also belong to the larger overarching whole.

In the case of common goals and interests, a clear interdependence can be seen: after all, the aim is to achieve a result which requires contributions from *both* groups. In their model, Gaertner and Dovidio [17] describe a common goal in terms of ‘interdependence’. In this respect, collaboration between human and veterinary healthcare professionals is the result of addressing common goals and interests [2, 3, 6, 11, 16]. This is in line with the social interdependence theory which argues that interdependence results from a common goal [27–31]. Mutatis mutandis, this altruistic goal was recently incorporated in the One Health initiative (16). This means that One Health, as a common goal, can be expected to lead to greater collaboration. This brings us to our first hypothesis:

*One Health as a common goal has a positive effect on collaboration between human and veterinary healthcare professionals.*

The process of classifying concerns the individual’s perception of the connection between – in this case – two groups of healthcare professionals: how an individual sees his group or subgroup within a larger whole and whether the individual perceives the existence of a single overarching group, multiple groups or subgroups, or no group at all [17]. As described earlier, One Health can affect how people see themselves as part of a larger, common whole and how they classify themselves within that larger whole. The Common Ingroup Identity theory distinguishes the following types of perception and reclassification: recategorization, decategorization and mutual differentiation [21]. These types are elaborated below.

### Recategorization

Perceived commonality and perceived common goals (overlap between groups and a stronger feeling of ‘us’ rather than ‘us and them’, recategorization) result in greater collaboration [22, 32]. That being said, the feeling of ‘us’ is not by definition limited to a single group: a person can possess multiple identities because he or she can be a member of multiple groups [33]. This means that in addition to classifying themselves in the veterinarians’ group, veterinarians could also classify themselves in the (overarching) group of healthcare providers [34]. We speak of recategorization when an overarching identity is perceived in which old groups are represented as a whole or in a new form, for example as a subgroup. Via the formation of a subgroup, collaboration between the two groups of healthcare professionals is further enhanced, for instance through awareness of a common goal. This brings us to our second hypothesis:

*The positive relation between common goal and collaboration is mediated by the partial effect of recategorization*.

### Decategorization

According to Gaertner and Dovidio [17], a common goal causes perceptions to be reclassified into changed perceptions, thus leading to greater collaboration. It may be expected that if healthcare professionals become aware of a common goal, there will be room to recognize the overlap with the other group of healthcare professionals. This type of development is also known as decategorization. If a certain situation is perceived as a form of decategorization, the emotional group connections become less important, so that there will be room for individuals to recognize shared identities. In turn, this will lead individuals to have more extensive contacts with other individuals, as a result of which prejudices will decrease and positive attitudes towards people in a different group can be developed [17]. This brings us to our third hypothesis:

*The positive relation between common goal and collaboration is mediated by the partial effect of decategorization*.

### Mutual differentiation

Brown and Wade [35] and Molleman, Broekhuis, Stoffels and Jaspers [36] conclude that if one wishes to stimulate collaboration, both groups must be able to retain their old identity. It is possible for the two groups to collaborate, but the researchers believe it is important that both groups continue to operate separately and that both fulfil a complementary role within the framework of their common goal. Such a structure, with interdependence and individual space for each group, will ultimately reduce prejudice and tension on either side [21, 37, 38]. This perceived commonality can then lead to greater collaboration [22, 32, 39].

A thorough understanding of interdependence and common endeavour has a psychological effect on interaction, interrelationships and collaboration: it leads to recognition, stimulation and interaction [27]. In the case of mutual differentiation as a social categorization perception, there is appreciation of the knowledge and expertise on the part of the other professional group. According to Gaertner and Dovidio [17], ‘there is a win-win situation which produces positive feelings and stereotyping towards the other group, while the individual’s own group can define its own profile’. In the case of recategorization and mutual differentiation, it is important in both cases that the original identity is not abandoned when collaboration takes place. Both groups will then be able to retain some autonomy within a common whole and they will not stray too far into each other’s territory [36].

Where the common goal (*in casu* One Health) is perceived as collaboration by mutual differentiation, a special focus lies on the importance of each of the groups with respect to their different qualities and expertise. Hewstone and Brown [40] state that collaboration should be focused on complementary knowledge and expertise. Collaboration will be triggered by paying attention to each other’s knowledge and expertise, as a function of the clarity of a common goal. This brings us to our fourth hypothesis:

*The positive relation between common goal and collaboration is mediated by the partial effect of mutual differentiation*.

### Knowledge sharing

Ives, Torrey and Gordon [41] argue that having a clear common interest leads to situations in which an exchange of knowledge can take place. In addition to what follows from the Common Ingroup Identity Model, it can be assumed that knowledge sharing leads to collaboration because individuals can use each other’s expertise [42–44]. Knowledge sharing between teams and groups improves performance and effectiveness [45–47]. Added value can be achieved by having professionals from different backgrounds learning and working together, thanks to the possibilities offered in terms of exchanges, the integration of knowledge and innovation. Advantages are particularly associated with the sharing of implicit knowledge and new insights [42]. Collaboration is promoted by knowledge transfer through informal or small-scale processes and lateral, social contacts [48, 49]. Kramer and Brewer [22] showed that individuals were particularly inclined to share knowledge with others within their own group, but also that they can be more reticent with more distant contacts. In that case, and especially in the case of One Health, it is important to address perceived distance; when others are perceived as less distant, they will have fewer reservations to collaborate within the framework of One Health. Finally, Holmes [50] demonstrates a positive connection between (continuing) knowledge sharing and mutual ties, trust within a group and collaboration [22, 32, 51]. This brings us to our fifth hypothesis:

*The positive relation between common goal and collaboration is mediated by the partial effect of knowledge sharing.*

## Methods

### Sample

Our study sample consisted of 368 respondents. By means of a digital newsletter from the professional organizations – the Royal Dutch Veterinary Association (KNMvD) and the Royal Dutch Medical Association (KNMG) – human and veterinary healthcare professionals were invited on a one-off basis to complete the questionnaire. The questionnaire was sent to 40,000 human and 5000 veterinary healthcare professionals. In addition to these professional associations, human and veterinary healthcare professionals were contacted via social media such as LinkedIn and Twitter.

A total of 595 healthcare professionals responded, 368 of whom completed the survey in full. Of the 368 respondents, 58 (16%) were human healthcare professionals; 310 (84%) were veterinary healthcare professionals. The low response rate demonstrated by the group of human healthcare professionals could be explained by their limited interest in the One Health topic [5]. An illustration of this can be found in the difference in attention paid to the topic in the professional body’s journals. During the last five years, One Health was mentioned only nine times in the weekly Dutch journal of Human healthcare professionals; in contrast, every monthly issue of the veterinary journal elaborated on the topic. Of all our respondents, 57% were female. The average age of the respondents was 44, and respondents had been working in the profession for an average of approximately 16 years. 87% of respondents were still active in clinical practice and 10% were working outside clinical practice. Of the healthcare professionals, 289 respondents (79%) had completed their studies in Utrecht. 12% of respondents had studied abroad, and of these more than 90% had studied in Belgium. Of the 368 respondents, 283 (77%) were members of the professional association, over 75% regularly read a professional journal in their own field, and fewer than 25% regularly read a professional journal in another field. Of the 58 human healthcare professionals, 90% had a specialization listed in the Dutch healthcare professions register (BIG). In the veterinary sector, the 310 respondents included 179 domestic animal veterinarians, 27 equine veterinarians, 76 farm veterinarians and 6 special animal veterinarians. These general figures tally with the figures obtained from the professional associations; there are no indications of bias.

### Measures

The variables were measured with a series of questions that had been compiled from various existing validated questionnaires. Many original items were adapted to the specific situation of human and veterinary healthcare professionals in order to ensure that the items were meaningful to them. The questionnaire took approximately 15 min to complete.

Apart from a number of open questions (related to age or the number of working years), all items were based on a Likert scale (1–7) and can be interpreted as continuous variables, thus following the fundamental Ordinary Least Square (OLS) principles. The seven-point scale was used to obtain greater dispersion and hence more nuance in the data [52]. Table 1 reports the general descriptives of the variables (mean, SD, alpha and correlations).

**Table 1:** Construct descriptive statistics, these constructs are created based on the valid items of the PLS-SEM model

Construct	Theoretical range	Actual range	Mean	SD	Reliability
Collaboration	1–7	1.00–7.00	4.37	1.37	0.90
Common goal	1–7	1.00–7.00	5.33	1.04	0.89
Recategorization	1–7	1.00–7.00	4.04	1.01	0.85
Decategorization	1–7	1.00–7.00	4.40	1.07	0.91
Mutual differentiation	1–7	1.00–7.00	5.80	0.95	0.94
Knowledge sharing	1–7	2.75–7.00	5.56	0.78	0.82

The dependent variable, *collaboration*, was based on Bock, Zmud, Kim and Lee [53] with a construct reliability score of 0.90, to which the one-item question on the perceived degree of overlap formulated by Schubert and Otten [54] was added (the OSIO – Overlap of Self Ingroup and Outgroup). An example of an item is ‘I collaborate when the opportunity arises’.

*Common goal* was based on Fisman and Laupland [2] and Kahn [3] and has a construct reliability score of 0.89. Participants were asked the following: “Thinking of possible collaboration between physicians and veterinarians, to what extent do you agree with the following statements?”. An example of an item is ‘I think more could be done to stimulate innovation in healthcare’.

*Recategorization* was measured with questions based on Edmondson [55] with a construct reliability of 0.85. An example of an item is ‘In the collaboration between physicians and veterinarians in general, it is possible to raise problems and difficult subjects in the collaboration’.

*Decategorization* was measured with a combination of items based on the instruments developed by Doosje, Ellemers and Spears [56] and Shamir, Zakay, Breinin and Popper [57]. An example of an item is ‘In the collaboration between physicians and veterinarians in general, it is considered important to make a lasting contribution to the collaboration’.

*Mutual differentiation* was measured with questions based on Berendsen, Benneker, Groenier, Schuling, Grol and Meyboom-de Jong [58] with a construct reliability of 0.94. An example of an item is ‘In the collaboration between physicians and veterinarians in general, there is appreciation of the expertise of the other professional group and a readiness to pursue contact on it’.

*Knowledge sharing* was based on Connolly and Kellaway’s study [59] with a construct reliability of 0.82. An example of an item is ‘In the collaboration between physicians and veterinarians in general, I am prepared to share specific professional knowledge (expertise) with the other professional group’.

### Analysis strategy

The study sample consisted of 368 respondents. This size is acceptable in view of the rule of thumb provided by Barclay, Higgins and Thompson [60], which suggests using ten times the maximum number of paths aiming at any construct in the outer model (this is not applicable as no formative constructs were used) and the inner model. All construct variables (Collaboration, Common Goal, Recategorization, Decategorization, Mutual Differentiation, and Knowledge Sharing) are reflective constructs.

For the outer model evaluation, internal consistency reliability and convergent validity were examined. The construct reliability scores ranged between 0.82 and 0.94, which was acceptable [61], and for the convergent validity the Average Variance Extracted (AVEs) of the constructs was also good [62]; this is included in Table 1. Secondly, indicator reliability was examined and all factor loadings were found to be higher than 0.6 and as such acceptable [63]. The construct and the factor loadings proved to be satisfactory for use in the analysis, although it can be said that the coefficients of the determinants are low – and that they are negligible in the case of Mutual Differentiation and Decategorization for collaboration. Finally, discriminant validity was checked, comparing the AVEs of the constructs with the inter-construct correlations [62]. Additionally, cross-loadings were checked. Evidence was found to exclude three items from Mutual Differentiation due to cross-factor loadings.

Partial Least Squares Path Modelling (PLS-SEM) was conducted with SmartPLS version 2.0 [64]. For the partial least square algorithm, the path weighting scheme was used, and the maximum number of iterations was set to 300. As stop criterion, 10^-5 was used. A uniform value of 1 was used as an initial value for each of the outer weights [65].

## Results and discussion

Table 2 shows the correlations between de different variables. Reliability and convergent validity of the measurement model was also confirmed by computing standardized loadings for indicators (Table 2) and Bootstrap t-statistics for their significance [66], see Table 3. For this bootstrapping, 5000 subsamples were used with a bias-corrected bootstrap testing for a two-tailed significance of 95%. The coefficient of determination was found to be moderate for Collaboration (*R* ^2^ = 0.42). The effect size of Common Goal on Collaboration (*f* ^2^ = 0.12), Recategorization on Collaboration (*f* ^2^ = 0.03) and Knowledge Sharing on Collaboration (*f* ^2^ = 0.04) can be considered small [67]. The effect sizes of Decategorization on Collaboration (*f* ^2^ = 0.00) and Mutual Differentiation on Collaboration (*f* ^2^ = 0.00) were negligible.

**Table 2:** Correlations between latent variables and square roots of average variance extracted, numbers shown in boldface denote the square root of the average variance extracted, correlations are significant at the 0.01 level (2-tailed)

	Construct	Mean	SD	1	2	3	4	5	6
1	Collaboration	4.37	1.37	**0.77**					
2	Common goal	5.33	1.04	0.58	**0.77**				
3	Recategorization	4.04	1.01	0.38	0.28	**0.86**			
4	Decategorization	4.40	1.07	0.41	0.36	0.63	**0.79**		
5	Mutual differentiation	5.80	0.95	0.30	0.36	0.15	0.29	**0.92**	
6	Knowledge sharing	5.56	0.78	0.51	0.61	0.24	0.40	0.34	**0.73**

**Table 3:** Coefficients of determination and predictive relevance

	R^2^ incl.	R^2^ excl.	1-R^2^ incl.	*f* ^2^
*f* ^2^ Common Goal - > Collaboration	0.42	0.35	0.58	0.12
*f* ^2^ Recategorization - > Collaboration	0.42	0.40	0.58	0.03
*f* ^2^ Decategorization - > Collaboration	0.42	0.42	0.58	0.00
*f* ^2^ Mutual Differentiation - > Collaboration	0.42	0.42	0.58	0.00
*f* ^2^ Knowledge Sharing - > Collaboration	0.42	0.40	0.58	0.04
	Q^2^ incl.	Q^2^ excl.	1-Q^2^ incl.	*q* ^2^
*q* ^2^ Common Goal - > Collaboration	0.24	0.20	0.76	0.05
*q* ^2^ Recategorization - > Collaboration	0.24	0.23	0.76	0.01
*q* ^2^ Decategorization - > Collaboration	0.24	0.24	0.76	0.00
*q* ^2^ Mutual Differentiation - > Collaboration	0.24	0.24	0.76	0.00
*q* ^2^ Knowledge Sharing - > Collaboration	0.24	0.2296	0.76	0.02

To obtain the *Q* ^2^ values as an indicator of the model’s predictive relevance, the blindfolding procedure was used, resulting in small predictive relevance for Collaboration (*Q* ^2^ = 0.24), Knowledge Sharing (Q^2^ = 0.19), and Mutual Differentiation (*Q* ^2^ = 0.11). The effect size for the predictive relevance of Common Goal on Collaboration was very small (*q* ^2^ = 0.05), as was Knowledge Sharing on Collaboration (*q* ^2^ = 0.02). Finally, the procedures outlined by Preacher and Hayes [68] were followed to examine multiple mediation effects.

With the help of multiple mediation models, it is possible to observe not only the direct effect of Common Goal on Collaboration, but also the mediation effects. The mediation effects are *a*_1_ *b*_1_ = .051 (through Recategorization), *a*_2_ *b*_2_ = .037 (through Decategorization), *a*_3_ *b*_3_ = .024 (through Mutual Differentiation), and *a*_4_ *b*_4_ = .167 (through Knowledge Sharing).

**Fig. 1:**
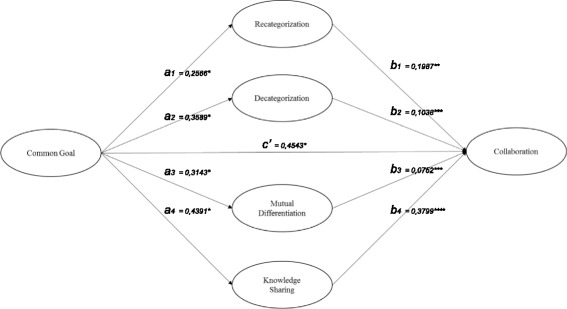
Mediation effects, *p = ≤ .001; **p = ≤ .008; ***p = ≥ .05; ****p = ≤ .0003

Figure 1 was designed based on the calculated mediation effects. All paths show a significant relation. This makes the Common Ingroup Identity Model an effective model to provide a plausible explanation why human and veterinary healthcare professionals do or do not collaborate. Having a common goal, like One Health, leads to collaboration via Recategorization. This mediating relation is also present for Knowledge Sharing. However, for Decategorization and Mutual Differentiation, there is a significant relation with Common Goal and with Collaboration; Decategorization and Mutual Differentiation have a direct relation with Collaboration that is not a mediating relation.

The PLS analysis confirms that a common goal promotes collaboration. Hypothesis 1 is therefore accepted. Bootstrapping the indirect effects of Common Goal on Collaboration, we found that Recategorization (0,051) and Knowledge Sharing (0,167) are significant mediators, thus supporting hypotheses 2 and 5. The specific indirect effect through Knowledge Sharing is larger than through Recategorization (effect Recategorization is significantly smaller; see contrasts) [68].

Significant relations were found between Common Goal via Decategorization with Collaboration and for Mutual Differentiation with collaboration. No evidence was found to support hypotheses 2 (mediating effect of decategorization) and 3 (mediating effect of mutual differentiation). Nevertheless, the results indicate an intervening effect for Decategorization and Mutual Differentiation, resulting in a satisfactory explanation why human and veterinary healthcare professionals do or do not collaborate. All four elements (Recategorization, Decategorization, Mutual Differentiation and Knowledge Sharing) are relevant; Recategorization and Knowledge Sharing are mediating variables.

Common Goal proved to be an important factor for promoting collaboration between human and veterinary healthcare professionals. This relationship is partly explained by the mediating role of recategorizing and knowledge sharing, but also partly by the intervening effects of Decategorization and Mutual Differentiation. Having a common goal (*in casu* One Health) produces altered perceptions in the form of an overarching identity. Upon recognizing their interdependence, human and veterinary healthcare professionals will initiate collaboration [27]. Collaboration, if there is a common goal, must therefore be seen to a greater extent as interdependence, as described earlier by Mohr and Spekman [69], in which there is still scope to retain individual identity, as argued by Brown and Wade [35] and Molleman et al. [36]. In the case of recategorization, there is scope to retain individual identity since an overarching identity is created. Gaertner and Dovidio [17] showed that a common goal leads to a reduction of prejudice and resistance between groups (thus influencing the perception of the situation), which in turn has an influence on collaboration. The current findings point in the same direction.

This study shows that in addition to recategorization, knowledge sharing also has a mediating role with respect to the influence of a common goal (One Health) on cooperation. Findings indicate that One Health stimulates knowledge sharing and in this way enhances collaboration between human and veterinary healthcare professionals. It may therefore be concluded that knowledge sharing is a promoting factor for human and veterinary healthcare professionals to use each other’s expertise [42–44]. The healthcare professionals have different backgrounds, but they improve their performance and make it more effective by learning from each other and by collaborating [45–47]. One Health is an important initiative to generate and promote knowledge sharing. This study has shown that knowledge sharing is in fact stimulated if the common goal of One Health is perceived as an invitation to work together on the basis of mutual interdependence. Seen in this way, continuous knowledge sharing will ultimately improve the ties and the forms of collaboration between the two groups of healthcare professionals [50].

This study has also shown that Decategorization has a significant effect in the relation between Common Goal and Collaboration, although this is not a mediating but an intervening effect. Our analyses show that this path, which has also been described by Gaertner and Dovidio [17], also applies to the human and veterinary healthcare professionals. Many healthcare professionals will be of the opinion that decategorization is not sufficiently concrete; they will therefore give it little importance, which might explain why we did not find a mediating effect.

This study has also revealed that Mutual Differentiation expresses a significant relation between Common Goal and Collaboration. However, as with Decategorization, there is no mediating effect between Collaboration and Common Goal for Mutual Differentiation. It may therefore be concluded that Mutual Differentiation helps to explain the collaboration between human and veterinary healthcare professionals. A possible explanation for the fact that no mediating effect was found for Mutual Differentiation may be limited insight in each other’s sectors and expertise, and any untapped added value still to be discovered [2].

### Practical implications

The current study’s findings indicate that in order to achieve greater collaboration between human and veterinary healthcare professionals, it is first and foremost necessary to define a common goal. That being said, the concept of One Health is not yet sufficiently ‘alive’ in the heads of healthcare professionals. Most of these professionals will probably not have a clear idea of how to interpret it, particularly in their own practice. Having a clear common goal will likely help them overcome the social dilemma that healthcare professionals face. After all, these professionals will only be triggered to work together once the common goal is shaped and starts to come alive, as is also argued by Ives, Torrey and Gordon [41]. These researchers state that having a clear idea of the goal stimulates knowledge sharing. The social dilemma among healthcare professionals referred to earlier [14] is thus overcome, and the added value of interaction becomes clear to them. A common goal (as investigated in this study) only comes into existence as a result of this concreteness, and this will trigger the mechanisms required to create an overarching identity [21].

Furthermore, it is of importance for both groups of healthcare professionals to know each other and to realize that they have a shared responsibility, not only in terms of combatting infectious diseases, but also in terms of providing optimum care for patients. This insight is expected to facilitate cooperation between the groups of healthcare professionals [40]. The notion that there are differences between the two groups does not necessarily imply that there is no, or could not be any, collaboration between them [17]. On the other hand, a word of caution is needed here: we have to be careful not to facilitate or create too much interference concerning the other professionals’ fields, because this could result in resistance [36].

Professional associations can play a facilitating role in creating a common goal, promoting recognition and fostering awareness with respect to common responsibilities. Knowledge sharing could be shaped, for example, by including articles from the other field in the professional journals of both professional groups. As respondents in the current study reported, fewer than 25% read a professional journal related to the other sector. The mutual inclusion of each other’s articles could be a first step in creating a relatively simple form of knowledge transfer. In addition to the publication of articles in each other’s professional journals, professional associations could offer joint interdisciplinary training programmes and refresher courses.

It has become increasingly clear that greater awareness of the added value of the common goal results in more extensive cooperation [21, 37, 38]. This awareness reveals not only what both groups of healthcare professionals have in common, but also that human and veterinary healthcare professionals have more in common than they themselves realize. This insight will lower the current psychological barriers between the two groups of healthcare professionals, resulting in more extensive collaboration between them [17]. This awareness among both groups of healthcare professionals could be further improved via the communications issued by their professional groups.

### Limitations and future research

As the current study was a cross-sectional study, it has certain limitations concerning long-term effects or relations. In order to gain a deeper insight into possible causes and consequences, longitudinal research is needed. One Health is an interdisciplinary approach and less concrete for healthcare professionals. To stimulate collaboration on the basis of the arguments presented in the current study’s introduction, additional and more detailed research is necessary: although One Health has been studied as an overarching concept, individual elements have been somewhat neglected. Beyond that, we recommend more qualitative research on this subject. This is needed to obtain greater insight not only into ‘physicians’ and veterinarians’ thoughts and feelings, but also into the overlaps between the two groups. In addition, an international study is needed to compare the different worlds. For instance, to the best of our knowledge, in the Western world veterinarians generally are greater all-rounders than physicians, but in the developing countries we see that physicians are also all-rounders; one would expect that the psychological barrier will be lower between these health care professionals. We expect this to have an influence on their cooperation.

## Conclusion

To the best of our knowledge, this study is the first research project in which the Common Ingroup Identity Model is quantitatively researched with the help of questionnaires. It is recommended that further research be conducted into this model with a view to using it in more quantitative analyses. Collaboration between healthcare professionals - the One Health approach - can be further investigated by focusing on other characteristics that influence the collaboration between the two groups. Possible options are stereotyping and social value orientation. The healthcare sector, and specifically the interaction between the human and veterinary fields, offers untapped potential, such as the development of treatments which could improve the healthcare as a whole.

Every study that analyzes the interaction between the two fields and addresses the healthcare professional’s social dilemma will be of immense value to society and can possibly give indications how to improve the quality of life. This study has shown that the Common Ingroup Identity Model helps to explain why human and veterinary healthcare professionals do or do not collaborate. Stimulating the interdependency perception, which can be reached with a clear common goal like the One Health approach, could increase collaboration between these health care professionals.

## Abbreviations

AVAs: Average variance extracted
BIG: Dutch healthcare professions register
CDCP: USA centres for disease control and prevention
GDP: Gross domestic product
KNMG: Royal Dutch Medical Association
KNMvD: Royal Dutch Veterinary Association
OLS: Ordinary least square
OSIO: Overlap of self ingroup and outgroup
PLS-SEM: Partial least squares path modelling
SARS: Severe acute respiratory syndrome
WHO: World Health Organization
